# Implementation of Antibiotic Stewardship Improves the Quality of Blood Culture Diagnostics at an Intensive Care Unit of a University Hospital

**DOI:** 10.3390/jcm11133675

**Published:** 2022-06-25

**Authors:** Sarah V. Walker, Benedict Steffens, David Sander, Wolfgang A. Wetsch

**Affiliations:** 1 Faculty of Medicine, University of Cologne, 50923 Cologne, Germany; joseph.steffens@uk-koeln.de (B.S.); david.sander@uk-koeln.de (D.S.); wolfgang.wetsch@uk-koeln.de (W.A.W.); 2 Institute for Medical Microbiology, Immunology and Hygiene, University Hospital of Cologne, 50935 Cologne, Germany; 3 German Centre for Infection Research (DZIF), 38124 Braunschweig, Germany; 4 Department of Anesthesiology and Intensive Care Medicine, University Hospital of Cologne, 50937 Cologne, Germany

**Keywords:** antibiotic stewardship, diagnostic stewardship, blood culture, bloodstream infection, bacteremia

## Abstract

**Background**: Bloodstream infections increase morbidity and mortality in hospitalized patients and pose a significant burden for health care systems worldwide. Optimal blood culture diagnostics are essential for early detection and specific treatment. After assessing the quality parameters at a surgical intensive care unit for six months, we implemented a diagnostic stewardship bundle (DSB) to optimize blood culture diagnostics and then reevaluated its effects after six months. **Material and Methods:** All patients ≥18 years old and on the ward were included: pre-DSB 137 and post-DSB 158. The standard quality parameters were defined as the number of blood culture sets per diagnostic episode (≥2), the rate of contamination (2–3%), the rate of positivity (5–15%), the collection site (≥1 venipuncture per episode) and the filling volume of the bottles (8–10 mL, only post-DSB). The DSB included an informational video, a standard operating procedure, and ready-to-use paper crates with three culture sets. **Results:** From pre- to post-interventional, the number of ≥2 culture sets per episode increased from 63.9% (257/402) to 81.3% (230/283), and venipunctures increased from 42.5% (171/402) to 77.4% (219/283). The positivity rate decreased from 15.1% (108/714) to 12.8% (83/650), as did the contamination rate (3.8% to 3.6%). The majority of the aerobic bottles were filled within the target range (255/471, 54.1%), but in 96.6%, the anaerobic bottles were overfilled (451/467). **Conclusions:** The implementation of DSB improved the quality parameters at the unit, thus optimizing the blood culture diagnostics. Further measures seem necessary to decrease the contamination rate and optimize bottle filling significantly.

## 1. Introduction

Infectious diseases have been a major contributor to morbidity and mortality in humans ever since history books existed. Severe systemic infections and sepsis are still associated with high morbidity and mortality [1], and increased treatment costs, significantly placing a burden on national health care systems [2]. The early detection of a culprit pathogen—especially in bloodstream infections—is essential for targeted antibiotic therapy and optimal patient outcomes. To achieve this, high standards in blood culture diagnostics are required [3,4]. A failure to obtain blood specimens in a patient with a bloodstream infection leads to prolonged antibiotic therapy using broad-spectrum antibiotics, which may contribute to the development of multidrug resistance, especially in Gram-negative germs [5,6].

Antibiotic stewardship is a multimodal-education strategy targeted to guide clinicians toward the correct use of antibiotic therapies. Diagnostic stewardship is an essential part of Antibiotic Stewardship programs concentrating on the diagnostic process. The goal is to reduce the broad and prolonged use of wide-spectrum antibiotics by using targeted antibiotic therapy, which otherwise contributes to the occurrence of multi-resistant microbes [7,8,9]. National and international guidelines recommend its implementation, especially in intensive care [10]. Furthermore, the recommended guideline use of the Tarragona strategy also requires identifying the pathogen to allow for targeted antibiotic therapy [11]. False-positive blood cultures, due to contaminations, also lead to prolonged hospital stays, significantly increasing the overall costs [12] and enhancement of antibiotic resistance [13]. 

The optimal blood culture diagnostics include consequent disinfectant measures and a minimum of two to three blood culture sets per diagnostic episode, as the detection rate increases 3–5% per mL of collected blood [14,15]. In this combined retrospective and prospective interventional study, we first assessed the quality parameters for the blood culture diagnostics conducted at a surgical intensive care unit at the University Hospital of Cologne, Germany, over a period of six months. We then implemented a diagnostic stewardship bundle (DSB) to optimize and standardize the blood culture diagnostics at this ward and monitored its effect on our pre-defined quality parameters for six months. The DSB included an instruction video and a new standard operating procedure in the form of informational interventions, pre-packed ready-to-use paper crates with a reminder checklist, three pairs of blood cultures, and order forms for the microbiological lab. 

## 2. Materials and Methods

**Ethics:**

The Ethics Committee of the University of Cologne (Head: Prof. Dr. R. Voltz; ID: 20-1279) approved this study on 20 August 2020. Patient consent was not required, as an indication for obtaining samples was always based solely on clinical decisions.

**Study registration:**

The study was then registered at the German Clinical Trials Register (ID: DRKS00022995; the registration was finalized on 2 September 2020).

**Setting:**

This study was performed at the Intensive Care Unit (ICU) “1.C” of the Department of Anaesthesiology and Intensive Care Medicine at the University Hospital of Cologne (UHC), Germany. The UHC is a university-based teaching hospital with 1500 overall patient beds and 120 ICU beds. The ICU “1.C” has 14 beds and mainly treats postoperative patients from abdominal surgery, trauma surgery, transplantation, and vascular surgery. 

**Materials:**

We introduced a diagnostic stewardship bundle (DSB) to this ward, consisting of:

- A standardized training video from a consultant microbiologist on the quality parameters of blood culture diagnostics and procedures, focusing on disinfectant measures and indications for blood culture diagnostics. The viewing was mandatory for all physicians working in the ICU;
- A new interdisciplinary standard operating procedure (SOP) on how to take blood cultures;
- Paper crates fitting three pairs of blood culture sets (SixBac, BD, Franklin Lakes, NJ, USA);
- A checklist as a reminder, highlighting essential information about blood cultures, which we glued to the crates.

**Figure 1:**
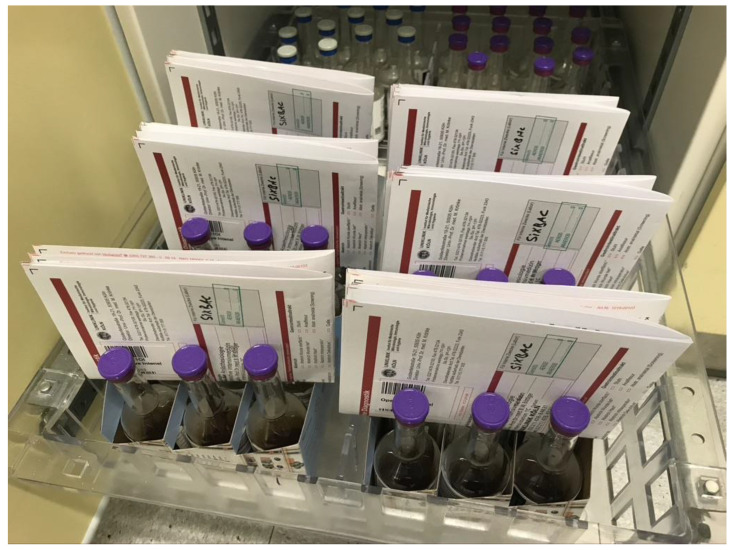
Ready-to-use “six-packs” of blood culture bottles pre-packed in paper crates with pre-filled microbiology lab order forms were implemented in the intensive care unit. On the side of the crates, we placed a sticker with a short summary of the most important points of the new standard operating procedure (SOP). The standard bottles were stored behind in case more than six bottles were needed.

The paper crates were kept stocked with ready-to-use blood culture sets and order forms for the microbiological laboratory (Figure 1). The standard of blood culture work was defined as a minimum of two blood cultures per episode, with at least one being withdrawn from a single venipuncture. If a patient had intravenous-access devices, one blood culture set per device was to be collected from them, as well.

**Methods:**

In the summer of 2020, we introduced the DSB, which consisted of staff training, the SOP, and pre-packed paper crates (see above) at the ICU 1.C. In a prospective way, we analysed all the blood culture results during the 6-month intervention period (September 2020 to February 2021) regarding the number of blood culture sets per episode, collection site, positivity, and contamination rate. The same months of the period, from September 2019 to February 2020 (6 months), were analysed retrospectively and served as a control group (pre-DSB period), as seen in Supplementary Figure S1.

The blood cultures of all adult patients (age ≥ 18 years) at the ICU were included. An episode was defined as all blood culture sets collected from one patient within 24 h. A blood culture set was defined as a pair of aerobic-resin and anaerobic-lytic blood culture media (Becton Dickinson (BD), Heidelberg, Germany).

From September 2020 to February 2021 (6 months), we implemented the diagnostic stewardship bundle (DSB) in this ICU and monitored its effect on the defined quality parameters. For those quality parameters, target ranges were defined as follows: ≥2–3 blood culture sets per episode, 5–15% positivity rate, 2–3% contamination rate, 8–10 mL blood-filling volume per bottle, and ≥1 venipuncture as the collection site per episode.

Additionally, the collected blood volume per bottle was derived by weighing each bottle. Beforehand, the empty blood culture bottles of the aerobic-resin and anaerobic-lytic blood culture (BD) media were weighed. Combined with the specific weight of the blood (set as 1.057 g per mL) [16], the target weight range of optimal blood-filling was defined as corresponding to 8–10 mL of blood per bottle, according to the manufacturer (aerobic: 68.36–71.2 g; anaerobic: 69.26–72.37 g).

The blood culture diagnostics in the microbiological laboratory were performed according to standard laboratory protocols: Each bottle was incubated in the BACTEC 9240 blood culture system (BD, Franklin Lakes, NJ, USA) for 1 week, and 2 weeks if endocarditis was suspected. Species identification was ensured by MALDI-TOF MS (Bruker Daltonic GmbH, Bremen, Germany).

According to the species identified, clinical assessment, and an indication for anti-infective therapy, the episodes were classified as “sterile”, “relevant”, or “contamination”. Typical contaminants were species of physiological skin flora, e.g., coagulase-negative staphylococci. In the case of repeated growth of typical contaminants and plausible clinical settings, they were classified as relevant episodes. 

The contamination rate was defined as the number of false-positive (contaminated) bottles from all collected blood culture bottles.

**Statistics**

Analysis of data was performed using IBM SPSS 28 (IBM, Armonk, NY, USA). Descriptive statistics were made using univariate analyses. Quality parameters of the pre-intervention and the post-intervention study period were compared using Fisher’s exact test.

## 3. Results

During the pre-DSB period, 402 episodes of blood culture collection with 714 blood culture sets were documented. In the pre-DSB period, the majority of blood culture episodes contained two blood culture sets (257/402, 63.9%) followed by only one blood culture (145/402; 36.1%). Only 33 episodes contained 3 or more blood culture sets (3: 21/402, 5.2%; 4: 11/402, 2.7%; 6: 1/402, 0.2%). Of the 402 episodes with a specified collection site, 231 (57.5%) consisted of the blood culture sets collected from central intravenous lines solely, whereas 171 episodes (42.5%) contained at least 1 blood culture set by peripheral venipuncture. Sterile episodes were of the majority (*n* = 329, 81.8%), followed by relevant (*n* = 51, 12.7%) and contamination (*n* = 22, 5.5%). In detail, growth was detected in 108 blood culture sets (positivity rate 15.1%) with a contamination rate of 3.8% (*n* = 27). The contamination rate of the blood cultures collected by peripheral venipuncture was 2.2% (5/223), and by the intravenous-access device, 4.6% (22/483); see Table 1. 

The main pathogenic species were *Enterococcus faecium* (36/78) followed by *Escherichia coli* (9/78), *Klebsiella pneumoniae* (6/78), *Enterobacter cloacae* (5/78), and *Candida albicans* (5/78). The predominant contaminant species was *Staphylococcus epidermidis* (17/30); see Supplementary Table S1.

During the intervention, 158 patients were included in this study, with 283 episodes and 647 blood culture sets. After introducing our DSB, 53 of 283 (18.7%) episodes consisted of one blood culture set, 106/283 (37.5%) of two, 114/283 (40.3%) of three, and 10/283 (3,5%) of four blood culture sets; see Table 1.

The classification of episodes did not differ significantly: sterile being predominant (*n* = 232, 82.0%), followed by relevant (*n* = 35, 12.4%) and contamination (*n* = 16, 5.7%). Neither did the positivity rate change significantly (83/650, 12.8%), nor the contamination rate (23/650, 3.6%), even though the latter decreased slightly. Neither did the contamination rate from the blood cultures collected by the peripheral venipunctures differ significantly (2.4%, 9/370; *p* = 1.000) from the pre-DSB period, nor did it from the blood cultures collected by an intravenous-access device (5.1%, 14/277; *p* = 0.8594); see Table 1.

The predominant pathogenic species were *Candida glabrata* (13/59), *Escherichia coli* (11/59), *Staphylococcus aureus* (11/59) and *Enterococcus faecium* (9/59). *Staphylococcus epidermidis* remained the main contaminant species (14/25); see Supplementary Table S1.

After the intervention, 77.4% (219/283) of the episodes included at least one blood culture collected from a separate venipuncture, which was a significant increase in comparison to the pre-interventional study period (*p* < 0.0001); see Table 1.

The majority of the contaminated blood culture bottles were collected from intravenous-access devices in both study periods (pre-DSB 77.8%, 21/27; post-DSB 60.9%, 14/23). However, even if they were not significant in the post-interventional period, the rate of the contaminated bottles collected from the intravenous-access devices decreased after implementing the DSB, from 21/27 pre-interventions to 14/23 post-interventions (*p* = 0.228); see Table 1.

An assessment of blood volume per bottle could only be performed during the intervention period and showed a broad variation between the aerobic and anaerobic bottles; Table 2. The majority of aerobic bottles (255/471, 54.1%) exhibited filling in the targeted range of 8–10 mL. Nearly one-third of the aerobic bottles (123/471, 26.1%) were overfilled, containing more than 10 mL of blood. Less than 8 mL of blood was found in 19.7% (93/471) of the aerobic bottles. The vast majority of anaerobic bottles, however, were filled with more than 10 mL of blood (451/467, 96.6%). Only 12 bottles (2.6%) showed an optimal filling volume and only 4 (0.9%) were filled with less than 8 mL of blood.

## 4. Discussion

In this combined retrospective and interventional prospective study, we assessed the quality of the status quo of blood culture diagnostics at the surgical intensive care unit “1.C” at the University Hospital of Cologne, implemented an diagnostic stewardship bundle (DSB), and monitored its effect on the above-mentioned quality parameters. To our best knowledge, this is the first study that systemically evaluates the effect of a combined DSB consisting of standardized training, the implementation of an SOP, and the use of pre-packed paper crates with blood culture bottles.

Diagnosing by blood culture is the gold standard for identifying pathogens in patients with sepsis or systemic infections [4]. Identifying a pathogen and determining antibiotic susceptibility are prerequisites for goal-directed antibiotic therapy. This may further help to reduce the widespread use of broad-spectrum antibiotics and thus the development of bacterial resistance against antibiotic agents. However, blood cultures may present as false-positive or false-negative, especially if not taken and treated appropriately. Appropriate amounts of blood must be drawn from representative locations and in adequate amounts to reduce the probability of false-negative tests, and contamination must be avoided to prevent false-positive results. Both false-negative and false-positive tests must be avoided, as they could have therapeutic consequences that harm the patient. Hence, the aim must be to achieve an optimal pre-test quality in order to obtain the highest benefit from this diagnostic test. 

The standard quality parameters were defined as the number of blood culture sets per diagnostic episode (two or more), the rate of contamination (2–3%), the rate of positivity (5–15%), the collection site (≥1 venipuncture per episode) and the filling volume of the bottles (8–10 mL) [3,14,15,17,18,19,20]. It has been well-established that the diagnostic sensitivity correlates to the collected number of blood culture sets per diagnostic episode: two blood culture sets have been described as the absolute minimum for adequate sensitivity, as three to four sets have been debated in the last years as the new standard [4,14] 

Before the implementation of the DSB, in 63.9% of the diagnostic episodes, two or more blood culture sets were collected—of which 55.7% contained a minimum of two bottles. The positivity rate was slightly higher than the defined maximum of 15%, indicating a tendency towards restrictiveness in blood culture collections. After the intervention, we were able to raise the number of episodes with two or more blood culture sets to 81.3%, at which 40.3% of the episodes contained even three sets, and in consequence, lessened the restrictiveness of blood culture collection, as indicated by the optimized positivity rate of 12.8%. As the detection rate increases by 3–5% per milliliter of blood, each additional set containing two bottles with 8–10 mL of blood may improve the clinical outcome of patients, due to optimized treatment options [15].

A blood culture collection from at least one separate venipuncture for each diagnostic episode is defined as state-of-the-art, as a collection from the intravenous-access devices is associated with higher rates of contamination by species of the physiological skin flora colonizing the devices, even if freshly placed [3,4]. In the pre-interventional period, almost every contaminated blood culture was collected from an intravenous-access device, supporting this thesis. After the intervention, our study did not find higher contamination rates of significance from intravenous-access devices, implying the success of the consequent disinfectant measures during the process. Additionally, in compliance with our DSB norm, the rate of venipuncture collections significantly increased by 34.9%, from 42.5% to 77.4% (*p* < 0.0001) after the intervention, improving the quality of the diagnostic process. However, the contamination rates from the blood cultures collected by peripheral venipuncture did not vary significantly between the two study periods; rather, they remained at a low level (2.2% to 2.4%).

Interestingly, before our intervention, the predominant pathogenic species was the *Enterococcus faecium* (*E. faecium*), which was detected 4–6 times more often than the second (*Escherichia coli*) and third (*Klebsiella pneumoniae*) most frequent species. After our intervention, the detected species shifted towards a more balanced species distribution, leaving *E. faecium* as the fourth most frequent species after *Candida glabrata*, *E. coli,* and *Klebsiella pneumoniae*. It is probable, however, that the species distribution was linked to a different patient cohort and not to our intervention. The study was conducted during the COVID-19 pandemic, which was a significant burden for all healthcare systems. With many patients requiring mechanical ventilation, there was a shortage of available ICU beds. In the control period (before COVID-19), there were mainly patients after solid organ transplantation, major abdominal surgery, and vascular surgery. During the intervention period (during the COVID-19 pandemic), almost no solid organ transplantations were made, and the postoperative surgical patients more often had undergone emergency surgery than elective procedures. In our opinion, this is the most probable reason for the change in the microbiological species isolates between the two time-frames. 

Contaminants mainly comprised coagulase-negative staphylococci and other typical species of the physiological skin flora in both study periods, even though Gram-negative species were especially detected in the pre-interventional period. This may be linked to the strict disinfectant measures during the collection process and fewer blood cultures from intravenous-access devices, which may be colonized by Gram-negative species, as well.

Our study revealed an optimization potential for the blood-filling volume of both aerobic and anaerobic media. The standardized schooling video and the standard operating procedure instructed to first fill the anaerobic bottle before filling the aerobic bottle. The vacuum in the anaerobic bottle may have led the blood culture collectors to overfill the bottles, as they may have underestimated the suction, thus stopping the filling process too late. It remains unclear if changing the order of the bottles in the filling process will reduce this effect or just shift the over-filling to the aerobic bottle. Additionally, optimizing the vacuum in the bottles for practical application may be a solution-oriented approach. As the pre-interventional part of the study was performed retrospectively, the blood-volume filling was not determinable for that study period. 

Our results implicate the importance of defining and surveilling quality parameters of blood cultures and implementing DSBs to improve the diagnostic process, even if the process is of a high standard already. The chosen bundle, with the combination of a schooling video that ensures standardized instructions for each staff member, the new standard operating procedure with an emphasis on when and where to take blood culture samples, and the pre-packed “six-pack” paper crates with a checklist summarizing the SOP, improved the quality of blood culture diagnostics. However, our results also show that the process still has to be optimized regarding the volume of the blood culture bottles, and a reduction in the contamination rate should also be targeted in future measures. 

One major limitation of our study is that it was conducted during the COVID-19 pandemic. Although this study was planned long before, the pandemic significantly delayed the start. The mandatory introduction was planned to be held in person; however, it had to be changed to a video format in order to avoid unnecessary person-to-person contact. Furthermore, the pandemic significantly changed the collective of patients treated in the regarded ICU, which is the most probable explanation for the shift in the identified pathogens.

## 5. Conclusions

In conclusion, our results show that the implementation of an diagnostic stewardship bundle in an intensive care unit has shown to be effective in improving blood culture diagnostics. The combination of a standardized schooling video (that every staff member had to watch), a standard operating procedure, and the introduction of pre-packed “six-packs” of blood culture bottles in paper crates helped to improve the sample quality, and especially the rate of the peripheral blood cultures drawn from venipuncture. However, further measures seem necessary to decrease the contamination rate and optimize bottle filling. 

## Figures and Tables

**Table 1:** Quality parameters of the pre-intervention and the post-intervention study period; DSB: diagnostic stewardship bundle.

	Pre-Intervention-Period		DSB-Intervention-Period		*p*-Value
Number of					
Patients included	137		158		
Episodes	402		283		
Blood culture sets	706		647		
Blood culture sets per episode					
1	145	36.1%	53	18.7%	<0.0001
2	224	55.7%	106	37.5%	<0.0001
3	21	5.2%	114	40.3%	<0.0001
4	11	2.7%	10	3.5%	0.2561
5	0	0%	0	0%	1.000
6	1	0.3%	0	0%	0.3832
At least 1 peripheral venipuncture	171	42.5%	219	77.4%	<0.0001
Only intravenous-access device	231	57.5%	64	22.6%	<0.0001
Collection site not specified	6	-	-	-	-
Positivity rate					
Number of blood culture sets with growth	108	15.1%	83	12.8%	0.2126
Sterile blood culture sets	606	84.9%	567	87.6%	
Blood culture sets with growth of a contaminant	27 *	3.8%	23 *	3.6%	0.8857
Blood culture sets with pathogenic species	81 *	11.3%	60 *	9.3%	0.2132
Contaminated blood culture sets collected by peripheral venipuncture	5	2.2%	9	2.4%	1.000
Contaminated blood culture sets collected by intravenous-access device	22	4.6%	14	5.1%	0.8594
Classification of episode					
Sterile	329	81.5%	232	82.0%	1.000
Relevant species	51	12.7%	35	12.4%	1.000
Contamination	22	5.5%	16	5.7%	1.000

* Some episodes contained both a contaminant and a pathogenic species.

**Table 2:** Blood volume of aerobic and anaerobic blood culture bottles at the post-interventional study period.

Bottle Volume	Aerobic		Anaerobic	
≥8–≤10 mL	255	54.1%	12	2.6%
<8 mL	93	19.7%	4	0.9%
>10 mL	123	26.1%	451	96.6%
Sum of weighed bottles	471		467 *	
Mean volume (mL)	9.15		13.37	

* Four blood culture sets were sent with only the aerobic bottle.

## Data Availability

The data presented in this study are available on request from the corresponding author. The data are not publicly available due to ethical reasons.

## Supplementary Materials

The following supporting information can be downloaded at: https://www.mdpi.com/article/10.3390/jcm11133675/s1, Figure S1: Graphical abstract of timeline and intervention tools of the study; Table S1: Species distribution of contaminant and pathogenic species of the pre-intervention and post-intervention period.
