# Supplementary material for: Implementation of Antibiotic Stewardship Improves the Quality of Blood Culture Diagnostics at an Intensive Care Unit of a University Hospital
Source: J Clin Med. 2022 Jun 25;11(13):3675. doi: 10.3390/jcm11133675 (PMC9267444; doi:10.3390/jcm11133675)
Supplement: Supplementary file 1 [file jcm-11-03675-s001.zip › jcm-1786456-supplementary.pdf]

Implementation of Antibiotic Stewardship improves the quality of blood culture diagnostics at an intensive care unit of a university hospital

Sarah V. Walker[1,2,3 *], Benedict Steffens[1,2], David Sander[1,4] and Wolfgang A. Wetsch[1,4]

Supplementententary Data:

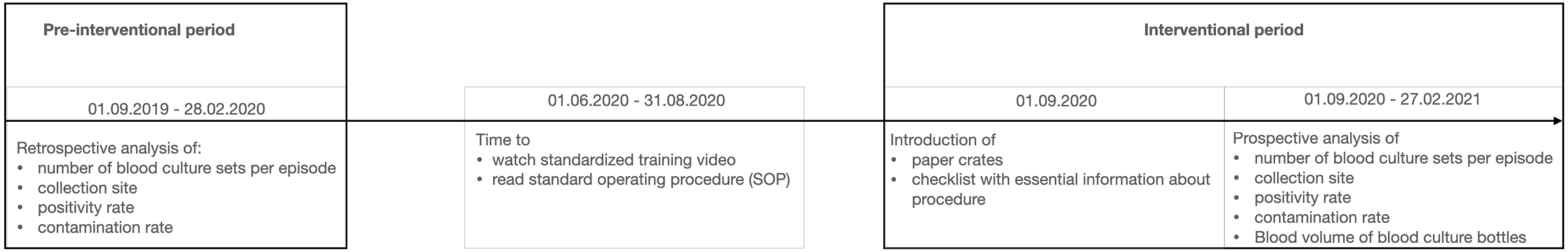

Supplementary Figure S1: Graphical abstract of timeline and intervention tools of the study.

Species	Pre-intervention	Post-intervention
Contaminant species	27	23
Bacillus cereus	0	1
Bacteroides uniformis	0	1
Corynebacterium spp.	1	0
Staphylococcus arlettae	0	1
Staphylococcus capitis	3	2
Staphylococcus haemolyticus	3	2
Staphylococcus hominis	2	2
Staphylococcus. epidermidis	17	14
Stenotrophomonas maltophilia	1	0
Pathogenic species	81	60
Acinetobacter dijkshorniae	1	0
Bacteroides cellulosilyticus	0	1
Bacteroides ovatus	2	0
Candida albicans	5	1
Candida dublinensis	2	0
Candida glabrata	2	13
Candida krusei	3	0
Candida spp. (not identified)	1	0
Enterobacter cloacae	5	0
Enterococcus faecalis	0	4
Enterococcus faecium	36	9
Escherichia coli	9	11
Klebsiella pneumoniae	6	4
Klebsiella spp. (not identified)	1	0
Klebsiella varicola	1	0
Pseudomonas aeruginosa	1	0
Staphylococcus aureus	1	11
Staphylococcus epidermidis	0	6
Staphylococcus lugdunensis	2	0
Streptococcus anginosus	2	0
Streptococcus constellatus	1	0

Supplementary Table S1: Species distribution of contaminant and pathogenic species of the pre-intervention and post-intervention period.
